# TELEPSICHOLOGYY AND OTHER CUTTING-EDGE TECHNOLOGIES IN COVID-19 PANDEMIC: BRIDGING THE DISTANCE IN MENTAL HEALTH ASSISTANCE

**DOI:** 10.1192/j.eurpsy.2023.1816

**Published:** 2023-07-19

**Authors:** I. S. Lancia, G. M. Festa, M. Mancini

**Affiliations:** 1 Interdisciplinary Institute of Higher Clinical Education (IAFeC); 2 Pontifical Faculty of Educational Sciences «AUXILIUM», Rome, Italy

## Abstract

**Introduction:**

Background: At the end of 2019, a novel coronavirus (COVID-19) was identified in China. The high potential of human-to-human transmission led to subsequent COVID-19 global pandemic. Public health strategies, including reduced social contact and lockdown have been adopted in many countries. Nonetheless, social distancing and isolation could also represent risk factors for mental disorders, resulting in loneliness, reduced social support and under-detection of mental health needs. Along with this, social distancing determines a relevant obstacle for direct access to psychiatric care services. The pandemic generates the urgent need for integrating technology into innovative models of mental healthcare.

**Objectives:**

In this paper, we discuss the potential role of Telepsichologyy (TP) and other cutting-edge technologies in the management of mental health assistance.

**Methods:**

We narratively review the literature to examine the advantages and risks related to the extensive application of these new therapeutic settings, along with the possible limitations and ethical concerns.

**Results:**

Telemental health services may be particularly flexible and appropriate for the support of patients, family members and healthcare providers during this COVID-19 pandemic. The integration of TP with other technological innovations (eg, mobile apps, virtual reality, big data and artificial intelligence (AI)) opens up interesting future perspectives for the improvement of mental health assistance.

**Conclusions:**

Telepsichologyy is a promising and growing way to deliver mental health services; but it is still underused. The COVID-19 pandemic may serve as an opportunity to introduce and promote, among numerous mental health professionals, the knowledge of the possibilities offered by the digital era.

**Disclosure of Interest:**

None Declared

